# A Novel Hybrid Clonal Selection Algorithm with Combinatorial Recombination and Modified Hypermutation Operators for Global Optimization

**DOI:** 10.1155/2016/6204728

**Published:** 2016-09-08

**Authors:** Weiwei Zhang, Jingjing Lin, Honglei Jing, Qiuwen Zhang

**Affiliations:** School of Computer and Communication Engineering, Zhengzhou University of Light Industry, Zhengzhou 450000, China

## Abstract

Artificial immune system is one of the most recently introduced intelligence methods which was inspired by biological immune system. Most immune system inspired algorithms are based on the clonal selection principle, known as clonal selection algorithms (CSAs). When coping with complex optimization problems with the characteristics of multimodality, high dimension, rotation, and composition, the traditional CSAs often suffer from the premature convergence and unsatisfied accuracy. To address these concerning issues, a recombination operator inspired by the biological combinatorial recombination is proposed at first. The recombination operator could generate the promising candidate solution to enhance search ability of the CSA by fusing the information from random chosen parents. Furthermore, a modified hypermutation operator is introduced to construct more promising and efficient candidate solutions. A set of 16 common used benchmark functions are adopted to test the effectiveness and efficiency of the recombination and hypermutation operators. The comparisons with classic CSA, CSA with recombination operator (RCSA), and CSA with recombination and modified hypermutation operator (RHCSA) demonstrate that the proposed algorithm significantly improves the performance of classic CSA. Moreover, comparison with the state-of-the-art algorithms shows that the proposed algorithm is quite competitive.

## 1. Introduction

Optimization techniques play a very important role in engineering design, commercial manufacture, financial market, information science, and related areas. An optimization problem can be expressed as

(1) Optimize fX,subject  to X∈Ω,

where *X* = [*x*_1_, *x*_2_,…, *x*_*D*_] is a* D*-dimensional vector of decision variables in the feasible region *Ω*. The optimization could be either a minimization problem or a maximization problem. Traditional optimization algorithms often fail to deal with multimodal, nonconvex, nondifferentiable problems since most of them rely on the gradient information [1]. In the past few decades, inspired by nature, people have developed many optimization computation methods to solve the complicated optimization problems, including genetic algorithm (GA), differential evolution (DE), particle swarm optimization (PSO) artificial immune system (AIS), and some new nature-inspired algorithms [2–7].

Among them, artificial immune system (AIS) is a newly emerging computational paradigm inspired by the fundamentals of immune system. It abstracts the structure and function of the biological immune system and exploits the applications to solve computational problems. In the area of optimization, numerical comparisons demonstrated that the performance of AIS is competitive compared to that of the other nature-inspired algorithms [8]. Among them, clonal selection algorithm is one of well-known AIS and has been applied to solve many practical optimization problems [9–11]. The clonal selection algorithm is inspired from behavior of B cells in secreting antibody to bind with the invading antigen. In view of the good performance of clonal selection algorithms (CSAs) in computational optimization area [12], in this paper, our work concentrates on CSA.

CSAs have attracted a lot of attention since they were developed [13–15]. However, with the increase of the complexity of the optimization problems, the deficiency of CSA is gradually exposed. One of the main shortages is that hypermutation is the main operator to modify the construction of the candidate solution in the traditional CSAs, and generally both global search and local search are based on adjusting the step size of the hypermutation. It would be barely unsatisfactory on balancing the diversity and convergence. In some improved versions, new randomly generated solutions are introduced by receptor editing or other mechanisms to increase diversity. However, the mechanism of randomly introduced solutions, along with the step-size controlled hypermutation leads to a blind optima searching process. Thereafter, premature convergence and diversity loss happen when dealing with the high-dimensional, multimodal optimization problems.

In view of the above analysis, we are motivated to explore the undeveloped potential of clonal selection theory in this paper. Inspired by the immune response of B cells, a combinatorial recombination operator is introduced to share the responsibility with hypermutation. In the proposed algorithm, recombination operator is proposed to enhance the search ability of the CSA. Moreover, the hypermutation operator is modified to generate more promising candidate solutions.

The rest of this paper is organized as follows. In Section 2, the general framework of CSA algorithm is presented, and the research works on improved CSAs in the field of numerical optimization are reviewed. In Section 3, the RHCSA algorithm is proposed based on the new introduced recombination and the modified hypermutation operators.  Section 4 presents and discusses the experimental results. Finally, the conclusion is drawn in Section 5.

## 2. CSA Framework

The AIS has become popular from the late 1990s. Several books, journals, and conference papers have been published in the past few years. de Castro and Timmis [16] depict the main models in AIS such as clonal selection, immune networks, and negative selection theories. More detailed review of AIS and their applications can be found in [15, 17–19]. Here we will give a brief literature review of the contemporary research efforts in clonal selection based algorithms in handling the numerical optimization.

### 2.1. Clonal Selection Algorithms (CSAs)

The main idea of clonal selection theory lies in the phenomenon where B cell reacts to invaded antigen through modifying the receptor called antibody. The general one, named CLOGNALG [20], is one of the representatives for clonal selection algorithms. There are mainly three operations involved, which are cloning, hypermutation, and selection.

The general framework of clonal selection algorithm for optimization is presented as follows.

*Framework of CSA*

**Step 1 (initialization).**

Randomly initialize antibody population.

**Step 2 (evaluation).**

Evaluate the objective values of the antibody population as their fitness.

**Step 3 (cloning).**

Generate copies of the antibodies.

**Step 4 (hypermutation).**

Mutate all the generated copies.

**Step 5 (selection).**

Select the one with highest fitness to survive.

**Step 6 .**

Repeat Steps 2–5 until a termination criterion is met.

The main features of the CSA framework are (i) the clone number, usually proportionally to the affinity of antibody with respect to the antigens; (ii) the mutation rate, normally inversely proportional to the affinity; and (iii) the absence of recombination operators (such as crossover in GAs). Those characteristics expose deficiencies when facing the high-dimensional, nonconvex multimodal, and multiobjective optimization problems. First, it is obvious that the cloning operator consumes high computational resource. Second, hypermutation is insufficient to bear the burden of balancing both the global search and the local search, which will lead to the premature convergence and unsatisfied accuracy. Third, the lack of consideration on interaction among individuals in the population may lead to the search missing global awareness, that is, overly searching one or some areas of the search space while leaving the others unvisited.

### 2.2. Improved CSAs

To overcome the abovementioned shortcomings, a bunch of improved algorithms based on CSA are proposed. The research works can be briefly classified as three categories.

#### 2.2.1. Modification of the Operators or Introduction of New Operators within the CSA Framework

Cutello et al. introduced a real-coded clonal selection algorithm for global optimization, involving the cloning operator, inversely proportional hypermutation, and aging operator [21]. Three versions of somatic contiguous hypermutation operator are analyzed and proven to have better performance than standard bit mutation to some types of optimization problem [22]. Khilwani et al. [23] proposed a fast clonal algorithm (FCA) which designs a parallel mutation operator comprising Gaussian and Cauchy mutation strategies. Lu and Yang [24] introduce the Cauchy mutation for the improved CSA (IMCSA). Two versions of immune algorithm named OPT-IMMALG01 and OPT-IMMALG combined with the clonal operator, M hypermutation operator, aging operator, and (*μ* + *λ*) selection operator based on binary-code and real-code representation, respectively, are discussed. The experimental results approve the effectiveness of the algorithms in handing the high-dimensional global numerical optimization problem [12]. Randomized clonal expansion strategy is proposed to solve high-dimensional global optimization problem in [25].

#### 2.2.2. Combine CSA with Immune Network Theory

Immune network theory considers that the immune cells have relations with each other and hereafter the cells, molecules, and other related substances construct a network. CAS combing with immune network theory is more suitable for handling multimodal function optimization.

The opt-AINet algorithm is an earlier version of CSA with artificial immune networks, which is proposed to solve multimodal continuous function optimization [26]. Uniform cloning operator, affinity-based Gaussian mutation, and similarity-based suppressor are proposed. To enhance the parameter adaptation, an improved adaptive Artificial Immune Network called IA-AIS is proposed, where affinity-based cloning operator, controlled affinity-based Gaussian mutation, and dynamic suppressor are introduced [27]. By imitating the social behaviors of animals, social leaning mechanism is introduced to the immune network; an algorithm called AINet-SL is proposed. The population is separated into elitist swarm (ES) and the common swarm (CS). Nonlinear affinity-based cloning, self-learning mutation, and social learning mutation are employed [28]. Based on the affinity measure, the candidate solution space is divided into the elitist space, the common space, and the poor space, and different hypermutation strategies are applied, respectively, in MPAINet [29]. Concentration-Based Artificial Immune Network, where the concentration of antibody is introduced to stimulate and maintain the diversity of the population, is applied to continuous optimization [30], combinatorial optimization [31], and multiobjective optimization [32].

#### 2.2.3. Hybrid CSA

CSA is also hybrid with PSO, DE, and other evolutionary strategies. Hill-climbing local search operator is combined with immune algorithm in [33]. Differential immune clonal selection algorithm (DICSA) is put forward by Gong et al. [34], where the differential mutation and differential crossover operators are introduced. Orthogonal initialization and neighborhood orthogonal cloning operator are proposed in an orthogonal immune algorithm (OIA) [35]. CSA with nondominated neighborhood selection strategy (NNIA) [36] was proposed for multiobjective optimization. Shang et al. [37] proposed an immune clonal algorithm (NICA) for multiobjective optimization problems, which makes improvements on four aspects in comparison with the traditional clonal selection computing model.

Baldwin effect is introduced into CSA and formulated the Baldwinian clonal selection algorithm (BCSA), which guides the evolution of each antibody by the differential information of other antibodies in the population [38]. Gong et al. [39] substitute the mutation in CSA with the local search technique in* Lamarckian *Clonal Selection Algorithm (LCSA) and adopt recombination operator and tournament selection operator for numerical optimization. An enhanced CSA, hybrid learning CSA (HLCSA) [40], is proposed by introducing two learning mechanisms: Baldwinian learning and orthogonal learning.

Although many improvements on immune inspired algorithm have been realized, the abovementioned shortcoming of artificial immune algorithm, such as premature convergence, high computational cost, and unsatisfied accuracy that can negatively affect the application of immune algorithm, remains a problem. The search ability of immune algorithm is limited when dealing with high-dimensional complex optimization with different characteristics. In this paper, we propose an improved CSA, by introducing a recombination operator and modifying the hypermutation operator to cope with the complex optimization problems with the characteristic high-dimensional, nonconvex, rotated, and composed optimization problems.

## 3. CSA with Combinatorial Recombination and Modified Hypermutation

### 3.1. Combinatorial Recombination

In immunology, the presence of both recombination and somatic mutation takes the responsibility for the diversification of antibody genes. The recombination of the immunoglobulin gene segments is the first step when the cells are first exposed to antigen. In the past few decades, recombination is mentioned more as crossover in GA. However, as depicted in [41], the recombination of immunoglobulin genes involved in the production of antibodies differs from the recombination (crossover) of parental genes in sexual reproduction. In the former, nucleotides can be inserted and deleted randomly from the recombined gene segments, while in the latter the genetic mixture is generated from parental chromosomes.

**Figure 1:**
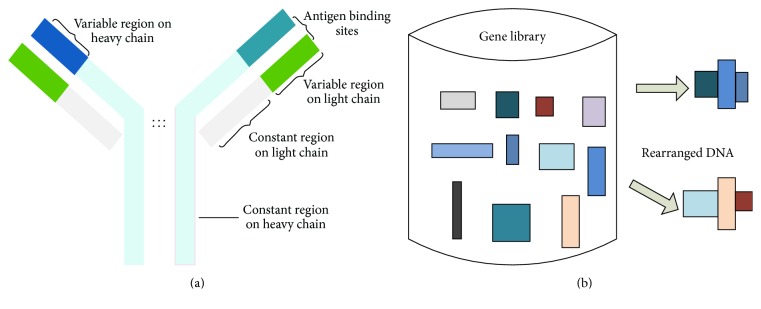
Structure of antibody (a) and recombination (b) in the variable region.

The basic unit of antibody molecule has a Y-shape structure, which contains two identical light chains and two identical heavy chains as shown in Figure 1(a). Variable regions located at the tips of the Y are primarily responsible for antigen recognition. Within these variable regions, some polypeptide segments show exceptional variability. The antibody molecules can be synthesized by an individual lying in the way of encoding the amino acid sequences of the variable domains into DNA chains as well as the random selection and recombination of gene segments as shown in Figure 1(b). During the development of B cell, the gene segments in the libraries are combined and rearranged at the level of the DNA. With the recombination of gene segments, recombination creates a population of cells that vary widely in their specificity. In this way, few immune cells are compatible with various antigens. After altering the base of antibody, the mutations fine-tune the lymphocyte receptor to better match the antigen [41, 42].

In the perspective of optimization, recombination functions as the coarse-grained exploration while hypermutation works the same way as fine-grained exploitation. Inspired by this, a combinatorial recombination operator is proposed as follows.

**Figure 2:**
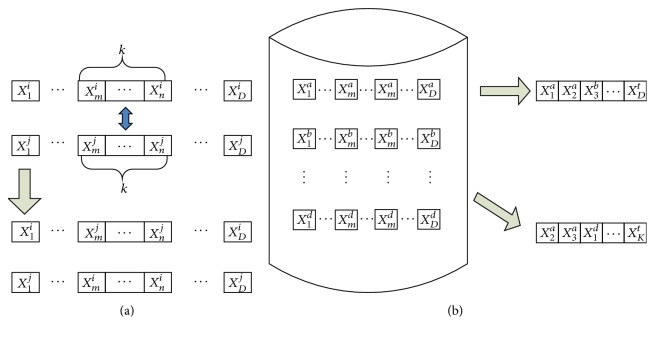
The way of rearrangement of gene segments (a) between two individuals (b) among several individuals.

To avoid the truncation error and the complexity of the coding, our algorithm is coded in real number and each dimension of a solution is viewed as a gene segment. The whole population forms the gene fragments library. According to the recombination in immunology, any orderly rearrangement of gene segments would generate a new B cell. As presented in Figure 2, the recombination could be (a) between two individuals as crossover or (b) among several individuals as the combination of randomly selected gene segments. With the help of normalization, the combination of gene segments could be in the specific order, such as in Figure 2(a) or can be randomly arranged as shown in Figure 2(b) in the computational respective.

The former one is similar to the SBX recombination in GA [42], where the crossover of parents could swap gene segments on one site or many sites. To better simulate the arrangement of gene segments, line recombination, DE inspired recombination [43], and intelligent recombination [44] are proposed. There would be a lot of ways to do the combinations with optional joined gene segments. With the randomness of the arrangements and combination, diversity is introduced. However, as is known to all, too much introduced diversity would be harmful for the performance. Based on the experimental experience, our work focuses on the way to process recombination between two parents. A new combinatorial recombination operator combining with line recombination is proposed as follows.

Randomly choose two individuals from the population, denoted by *X*_*A*_ and *X*_*B*_, and then randomly choose *m* dimensions, *m* ∈ [1, *D*], from each of them, where dimensions index could be recorded as vectors *V*_*A*_ and *V*_*B*_, respectively. The new individuals are generated by

**Figure 3:**
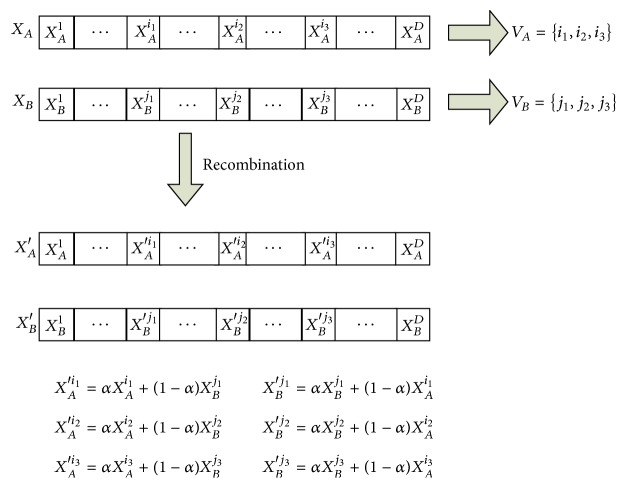
Recombination process.

(2) XA′VA=αXAVA+1−αXBVBXB′VB=αXBVB+1−αXAVA,

where *α* is a randomly produced number between 0 and 1. It should be noted that the range of each dimension of decision variable should be normalized at first. The new proposed recombination operator is represented in Figure 3.

As shown in Figure 3, two new individuals are generated through the combinational recombination. In the example, *m* equals 3. It needs to be known that *i*_1_ could be different from *j*_1_, the same as in *i*_2_ and *j*_2_ and *i*_3_ and *j*_3_ as long as the normalization has been done.

Then, the fitness of the new generated individuals is evaluated. Together with the original individuals, two with the higher fitness will survive, and the other two individuals are deleted; that is, choose two individuals with high fitness from the set {*X*_*A*_, *X*_*B*_, *X*_*A*_′, *X*_*B*_′}. Instead of comparing with the whole population and reserving the elite, a better diversity will be maintained this way.

### 3.2. Modified Hypermutation

Hypermutation operator brings diversity for the population by introducing perturbation for each clone. Although there are several ways to implement this operator [22], inversely proportional strategy is always the main basis. The concept of the operator proposed in [12] is adopted in this research work, where each candidate solution is subject to* M* mutations without explicitly using a mutation probability. The inversely proportional law is used to determine the number of the mutations* M*:

(3) α=exp⁡−ρf∗XiM=α×n+1,

where *f* ^*∗*^(*X*_*i*_)∈[0,1] is the normalized fitness of *X*_*i*_, *ρ* is the decay constant which determines the shape of the mutation rate, and ⌊·⌋ returns the lower bound integer. Then, *M* mutation is performed on each candidate solution:

(4) $$\begin{array}{c} X_{i}^{\prime j}=\left\{\begin{array}{cc} X_{r1}^{j}+\lambda \left(X_{r1}^{j}-X_{r2}^{j}\right) & \text{if }j\in \text{rand }M\left(n\right)\\ X_{i}^{j} & \text{otherwise} \end{array}\right.. \end{array}$$

*X*_*i*_(*j*) is the *j*th dimension of the *i*th individual, rand  *M*(*n*) ∈ {1,…, *n*} is randomly chosen *M* indexes without repetition, and *λ* is a random number in the range of [−1, 1]. *r*1, *r*2 ∈ {1,2,…, *N*} are randomly selected numbers; hereafter, the amplitude of the hypermutation is controlled automatically by the difference of randomly selected individuals in the population. The mutation equation (4) could be considered as the variant of differential evolution and is introduced by [45] to modify the original updating food source of artificial bee colony algorithm (ABC), where it is improved to benefit for enhancing the search ability of ABC. In our proposed algorithm, the equation is combined with the hypermutation inversely proportional* M* strategy as depicted above. The* M* strategy controls the direction, that is, along how many dimensions, while the equation controls the distance of the mutated clones with their parents. With combination of both, the amplitude of the hypermutation is automatically controlled according to the distribution of the population.

### 3.3. Framework of the Proposed Algorithm

Combined with the proposed combinatorial recombination and hypermutation operator, the framework of the proposed algorithm is as follows.

**Step 1 (initialization).**

Randomly initialize a population *Ab* of *N* individuals, where *N* denotes the size of the initial population. Each initial solution *X*_*i*_ = {*X*_*i*_(1), *X*_*i*_(2),…, *X*_*i*_(*D*)} is produced randomly within the range of boundaries of the decision space:

(5) $$\begin{array}{c} X_{i}^{j}=X_{min}^{j}+\text{rand }\left(\;0,1\;\right)\left(\;X_{max}^{j}-X_{min}^{j}\;\right), \end{array}$$

where *i* = 1,2,…, *N*, *j* = 1,2,…, *D*, where *D* is the dimension of the decision variables, and *X*_min_^*j*^ and *X*_max_^*j*^ are the lower and upper bounds for the dimension *j*, respectively.

**Step 2 (evaluation).**

Evaluate the objective value of each solution as its fitness.

**Step 3 (recombination).**

Randomly choose two individuals from the population and implement the combinatorial recombination as described in Section 3.1. The recombination rate is set to *N*_*r*_.

**Step 4 (clonal selection).**

(i) Cloning: each individual *X*_*i*_ generates *N*_*c*_ copies {*X*_*i*_^1^, *X*_*i*_^2^,…, *X*_*i*_^*N*_*c*_^}, where *N*_*c*_ is the clone number, which is a user defined constant.

(ii) Hypermutation: each clone *X*_*i*_^*j*^, *j* = 1,…, *N*_*c*_, goes through the hypermutation as described in Section 3.2 and generates the hypermutated clones *X*_*i*_^′*j*^.

(iii) Selection: select the individual with highest fitness among *X*_*i*_ and hypermutated clones {*X*_*i*_^′1^, *X*_*i*_^′2^,…, *X*_*i*_^′*N*_*c*_^}.

**Step 5 .**

If stopping condition is not met go to Step 3. Otherwise, output the best one of the feasible group.

It could be observed that instead of being proportional to the fitness, the clone number is a constant, which not only saves computational source but also avoids individuals being concentrated in some decision area and leaving the others unvisited when handling the multimodal problems.

## 4. Experimental Studies on Function Optimization Problems

In this section, experiments are conducted to evaluate the performance of RHCSA by using 16 commonly used global optimization benchmark problems. These functions contain the characteristics of being unimodal as *f*_1_~*f*_2_, unrotated multimodal as *f*_3_~*f*_8_, rotated multimodal as *f*_9_~*f*_14_, and composite functions as *f*_15_~*f*_16_.  Table 1 gives the expression of the benchmark function. The detailed characteristics of these functions can be found in [1, 46].

The introduced parameters are analyzed at first, and then the proposed algorithm is compared with traditional CSA in the benchmark functions. Finally, comparisons between the proposed algorithm and the state-of-the-art evolutionary computing models are represented. Some discussions on the performance analysis of RHCSA are also included. It needs to be noted that the same setup for the experiments is used for all the involved peers algorithms; that is, the comparison of all the algorithms is under the same configuration of experiment setup in this paper.

### 4.1. Experimental Parameters Settings

The experiments were conducted on 16 benchmark functions. The maximum number of function evaluations (mFES) is set as 10,000*∗D* for 10-*D* and 30-*D* situations, respectively. Each test is run 30 independent times.

There are quite few parameters introduced in the proposed algorithm. For a fair comparison among CSAs, they are tested using the same setting of the parameters, that is, the population size *N* is set to 30 and clone number *N*_*c*_ is set to 4 as in [40]. Furthermore, there are two new parameters introduced. One is *m* which controls the direction of combinatorial recombination, and the other is recombination rate. Tables 2 and 3 give the experimental results of varying *m* in the 16 benchmark functions with 10-*D* and 30-*D*. For convenience, the experiment sets 4 proportions to the dimension as the value to *m*, which are [⌈(1/10)*D*⌉, ⌈(1/5)*D*⌉, ⌈(1/3)*D*⌉, *D*].

The recombination operator could introduce diversity to the population through fusing information between randomly chosen parents. When *m* is small, the generated offspring is much similar to one of the chosen parents, and only little dimension obtains information from both parents. When *m* is large, the generated offspring tend to be the fusion of chosen parents. That is to say,* m* controls the offspring being much like one of the chosen parents or the fusion of both of them. From Tables 2 and 3, we can find that the difference among the varying setup of* m* is quite small. In a more careful observation, ⌈(1/5)*D*⌉ and ⌈(1/3)*D*⌉ are more appropriate to compare with the other situations for both* D* = 10 and* D* = 30, and ⌈(1/3)*D*⌉ is even better. It could be observed that experimental results are not sensitive to the parameter *m*, and a slightly larger *m* may be beneficial for the algorithm. In view of the observation,* m* = ⌈(1/3)*D*⌉ is chosen in our experiments.

There is another parameter named recombination rate *N*_*r*_, which controls the rate of recombination process, which will be implemented. The higher the *N*_*r*_, the more recombination processes that will be executed. In the early stage, the population is uniformly distributed in the search space, and recombination of randomly chosen individuals will bring diversity to the population. As is known to all, diversity is beneficial for enhancing the search ability of the algorithm, but too much diversity will slow the convergence. Tables 4 and 5 represent the experimental results of varying the recombination rate.

From Tables 4 and 5, it could be observed that RHCSA has a quite robust performance with the varying recombination rate. By comparison, *N*_*r*_ which is equal to 0.7 achieves the best performance and is adopted in the paper.

### 4.2. Comparisons with Classic CSA

The experiments are implemented to check if the proposed operators are beneficial for the performance of the algorithm. Firstly, recombination operator is added to the original CSA denoted as RCSA, and then the modified hypermutation is introduced as RHCSA.

Table 6 shows the statistical results of CLONALG, RCSA, and RHCSA in optimizing the 16 test problems with* D* = 10 based on 30 independent runs including the mean and standard deviation. From the table, we can observe that, for all these test instances, the introduced recombination operator could obviously improve the performance of the CSA. The reason is that the recombination operator could bring diversity to enhance the search ability and avoid being trapped into the local optima. The experiments results on multimodal function *f*_3_~*f*_8_ could present the ability of introduced recombination operator. With the modified hypermutation operator, the performance of the algorithm obtains a further improvement. This is because both recombination and hypermutation adopt the difference of chosen individuals to generate the candidate solution in the proposed algorithm. In the early stage, the population is distributed in the search space, and the difference is relatively large which is beneficial for global search and then, with the iterations going on, the population converges to the optima and the difference between individuals becomes smaller and smaller; that is, the search process turns to be a local search. It can be observed that RCSA surpass CSA and be surpassed by RHCSA at all the tested functions. It can be concluded that the proposed recombination and hypermutation operators are effective improving the ability of the CSA.

Table 7 shows the experimental results of the algorithms when *D* = 30. Similar results are represented, which indicates that RHCSA is able to handle the high-dimensional optimization problems as well.

### 4.3. Comparisons with the State-of-the-Art Algorithms

To compare RHCSA with the state-of-the-art algorithms, experimental results of seven representative evolutionary algorithms are listed in Tables 8 and 9. These algorithms are Baldwinian clonal selection algorithm (BCSA) [38]; hybrid learning clonal selection algorithm (HLCSA) [40]; orthogonal crossover based differential evolution (OXDE) [47]; self-adaptive differential evolution (SaDE) [48]; global and local real-coded genetic algorithm (GL-25) [49]; and comprehensive learning particle swarm optimization (CLPSO) [1].

Table 8 presents the mean values and standard deviations of the seven algorithms on the 16 test functions with *D* = 10, where the best results are shown in bold face. First of all, RHCSA performs the best on the 6 unrotated multimodal functions *f*_3_~*f*_8_. It could exactly locate the global optima in 7 functions, *f*_4_~*f*_8_, *f*_11_, and *f*_15_. In particular, it is observed that RHCSA surpasses all the other algorithms on functions *f*_3_ and *f*_12_ with great superiority. Moreover, RHCSA, HLCSA, BCSA, SaDE, and CLPSO can obtain the global minima 0 on functions *f*_5_, *f*_6_, *f*_7_, and *f*_8_; RHCSA, HLCSA, BCSA, SaDE, and OXDE get the global optimum of function *f*_11_ and RHCSA, HLCSA, SaDE, OXDE, and GL-25 find the optimum on function *f*_15_. Though RHCSA cannot get the best results on functions *f*_1_, *f*_9_, *f*_10_, *f*_13_, and *f*_16_, it still gets the comparable solutions with respect to the best results obtained by the other algorithms. To the composite problems *f*_15_, RHCSA reaches the global best as HLCSA, OXDE, SaDE, and GL-25. To the function *f*_16_, RHCSA surpasses the other algorithms except for HLCSA.

Similar results can be observed from Table 9 when *D* equals 30. RHCSA can consistently obtain good results which are even slightly better than those on 10-*D* problems. RHCSA achieves the best performance on functions *f*_3_, *f*_4_, *f*_5_, *f*_6_, *f*_7_, *f*_8_, *f*_10_, *f*_11_, *f*_15_, and *f*_16_ compared with the other algorithms. Even RHCSA cannot find the best results on functions *f*_9_, *f*_12_, and *f*_13_; its obtained results are very close to the best results obtained by the other algorithms. For example, the best result on function *f*_12_  (1.6549*e* + 001 ± 4.4609*e* + 000) is found by OXDE while a comparable result obtained by RHCSA is 2.6201*e* + 001 ± 8.3454*e* + 000. RHCSA greatly improves the results on functions *f*_3_, *f*_14_, and *f*_16_. It obtains comparatively good results on functions *f*_9_, *f*_12_, and *f*_13_. It can be observed that the scalability of RHCSA is pretty well when dealing with high-dimensional optimization problems.

## 5. Conclusion

This paper presents a new clonal selection based algorithm, in which combinatorial recombination and hypermutation are introduced to enhance the search ability. The proposed algorithm is tested on 16 commonly used benchmark functions with unimodal, multimodal, rotation, and composite characteristics. Firstly, the CSAs with and without recombination operator is compared. The experimental results indicate that the proposed recombination operator is able to improve the performance of the CSA. And then the modified hypermutation makes further improvement. Finally, the proposed algorithm is compared with the state-of-the-art algorithms. The experimental results show the competitiveness of the RHCSA.

## Figures and Tables

**Table 1:** Benchmark functions used in our experimental study.

Name	Test function	*D*	*S*	*f*_min_
Sphere function	$f_{1}\left(x\right)=\overset{D}{\underset{i=1}{\sum }}x_{i}^{2}$	10/30	[−100,100]	0
Rosenbrock's function	$f_{2}\left(x\right)=\overset{D-1}{\underset{i=1}{\sum }}\left(100{\left(x_{i}^{2}-x_{i+1}\right)}^{2}+{\left(x_{i}-1\right)}^{2}\right)$	10/30	[−2.048,2.048]	0
Ackley's function	$f_{3}\left(x\right)=-20\;exp\left(-0.2\sqrt{\frac{1}{D}\overset{D}{\underset{i=1}{\sum }}x_{i}^{2}}\right)-exp\left(\frac{1}{D}\overset{D}{\underset{i=1}{\sum }}\left(2\pi x_{i}\right)\right)+20+e$	10/30	[−32.768,32.768]	0
Griewanks's function	$f_{4}\left(x\right)=\overset{D}{\underset{i=1}{\sum }}\frac{x_{i}^{2}}{4000}-\prod_{i=1}^{D}\cos\frac{x_{i}}{\sqrt{i}}+1$	10/30	[−600,600]	0
Weierstrass function	$f_{5}\left(x\right)=\overset{D}{\underset{i=1}{\sum }}\left\{\overset{kmax}{\underset{k=1}{\sum }}\left[a^{k}\cos\left(2\pi b^{k}\left(x_{i}+0.5\right)\right)\right]\right\}-D\overset{kmax}{\underset{k=1}{\sum }}\left\{a^{k}\cos\left(2\pi b^{k}\cdot 0.5\right)\right\}$	10/30	[−0.5,0.5]	0
	*a* = 0.5, *b* = 3, *k*max = 20			
Rastrigin's function	$f_{6}\left(x\right)=\overset{D}{\underset{i=1}{\sum }}\left\{x_{i}^{2}-10\cos\left(2\pi x_{i}\right)+10\right\}$	10/30	[−5.12,5.12]	0
Noncont.Ras	$f_{7}\left(x\right)=\overset{D}{\underset{i=1}{\sum }}\left\{y_{i}^{2}-10\cos\left(2\pi y_{i}\right)+10\right\}$, $y_{i}=\left\{\begin{array}{cc} x_{i} & \left|x_{i}\right|<0.5\\ \frac{round\left(2x_{i}\right)}{2} & \left|x_{i}\right|\geq 0.5 \end{array}\right.,\;\;i=1,2,\ldots ,D$	10/30	[−5.12,5.12]	0
Schwefel's function	$f_{8}\left(x\right)=418.9829D-\overset{D}{\underset{i=1}{\sum }}\left\{x_{i}\;\sin\left({\left|x_{i}\right|}^{0.5}\right)\right\}$	10/30	[−500,500]	0
Rot.Ackley's function	*f*_9_(*x*) = *f*_3_(*y*), *y* = *M∗x*	10/30	[−32.768,32.768]	0
Rot.Griewanks's function	*f*_10_(*x*) = *f*_4_(*y*), *y* = *M∗x*	10/30	[−600,600]	0
Rot.Weierstrass function	*f*_11_(*x*) = *f*_5_(*y*), *y* = *M∗x*	10/30	[−0.5,0.5]	0
Rot.Rastrigin's function	*f*_12_(*x*) = *f*_6_(*y*), *y* = *M∗x*	10/30	[−5.12,5.12]	0
Rot.noncon Ras function	*f*_13_(*x*) = *f*_7_(*y*), *y* = *M∗x*	10/30	[−5.12,5.12]	0
Rot.Schwefel's function	$f_{14}\left(x\right)=418.9829D-\overset{D}{\underset{i=1}{\sum }}Z_{i}$, $Z_{i}=\left\{\begin{array}{cc} y_{i}\sin\left({\left|y_{i}\right|}^{0.5}\right) & \left|y_{i}\right|\leq 500\\ 0.001{\left(\left|y_{i}\right|-500\right)}^{2} & \left|y_{i}\right|\geq 500 \end{array}\right.,\;i=\;1,2,\ldots ,D;\;\;$ *y* = *M* *∗* (*x* − 420.96) + 420.96	10/30	[−500,500]	0
Composition 1	*f*_15_ = *CF*1	10/30	[−5,5]	0
Composition 2	*f*_16_ = *CF*2	10/30	[−5,5]	0

**Table 2:** Results (mean ± std) of RHCSA with varying sampling points of combinatorial recombination in 16 benchmark functions with *D* = 10.

Function	*m* = ⌈(1/10)*D*⌉	*m* = ⌈(1/5)*D*⌉	*m* = ⌈(1/3)*D*⌉	*m* = *D*
*f*_1_	1.3521*e* − 180 ± 0.0000*e* − 000	1.53784*e* − 166 ± 0.0000*e* − 000	7.2585**e** − 195 ± 0.0000**e** − 000	2.2773*e* − 078 ± 4.9085*e* − 078
*f*_2_	8.6187*e* − 004 ± 3.20378*e* − 004	8.1377*e* − 004 ± 3.0271*e* − 004	8.9516*e* − 004 ± 3.2039*e* − 004	2.8971**e** − 004 ± 3.6648**e** − 004
*f*_3_	5.2831*e* − 015 ± 4.2156*e* − 015	1.7763*e* − 015 ± 1.7763*e* − 015	8.8817**e** − 016 ± 0.0000**e** − 000	2.0724*e* − 015 ± 2.0511*e* − 015
*f*_4_	0 ± 0	0 ± 0	0 ± 0	0 ± 0
*f*_5_	0 ± 0	0 ± 0	0 ± 0	0 ± 0
*f*_6_	0 ± 0	0 ± 0	0 ± 0	0 ± 0
*f*_7_	0 ± 0	0 ± 0	0 ± 0	0 ± 0
*f*_8_	0 ± 0	0 ± 0	0 ± 0	0 ± 0
*f*_9_	4.0175*e* − 015 ± 1.3376*e* − 015	3.0923**e** − 015 ± 1.4969**e** − 015	3.9292*e* − 015 ± 1.1420*e* − 015	4.2912*e* − 015 ± 1.0827*e* − 015
*f*_10_	9.3401*e* − 002 ± 1.5480*e* − 002	1.8195*e* − 002 ± 1.0245*e* − 002	1.6937**e** − 002 ± 1.6091**e** − 002	5.5463*e* − 002 ± 2.6635*e* − 002
*f*_11_	0 ± 0	0 ± 0	0 ± 0	0 ± 0
*f*_12_	2.4401*e* + 000 ± 1.0935*e* + 000	2.0556*e* + 000 ± 1.1601*e* + 000	1.5954**e** + 000 ± 1.5954**e** + 000	1.6638*e* + 000 ± 1.7026*e* + 000
*f*_13_	8.1024*e* + 000 ± 5.6678*e* + 000	4.1385*e* + 000 ± 2.1123*e* + 000	4.0000**e** + 000 ± 2.1213**e** + 000	4.1773*e* + 000 ± 3.7530*e* + 000
*f*_14_	7.5454*e* − 008 ± 6.2091*e* − 007	6.8712**e** − 008 ± 7.0921**e** − 007	9.8137*e* − 008 ± 7.7851*e* − 007	9.2565*e* − 008 ± 8.6635*e* − 007
*f*_15_	0 ± 0	0 ± 0	0 ± 0	0 ± 0
*f*_16_	9.0023*e* + 000 ± 7.8901*e* + 000	6.4971*e* + 000 ± 7.9297*e* + 000	5.2613**e** + 000 ± 5.0573**e** + 000	8.6977*e* + 000 ± 7.4630*e* + 000

**Table 3:** Results (mean ± std) of RHCSA with varying sampling points of combinatorial recombination in 16 benchmark functions with *D* = 30.

Function	*m* = ⌈(1/10)*D*⌉	*m* = ⌈(1/5)*D*⌉	*m* = ⌈(1/3)*D*⌉	*m* = *D*
*f*_1_	3.1129*e* − 114 ± 4.4031*e* − 114	8.2698*e* − 110 ± 0.0000*e* − 000	1.6994**e** − 194 ± 0.0000**e** − 000	6.6159*e* − 108 ± 0.0000*e* − 000
*f*_2_	1.9281*e* − 007 ± 8.9376*e* − 007	7.4820*e* − 007 ± 3.0968*e* − 007	2.6862*e* − 007 ± 6.0065*e* − 007	7.4820**e** − 008 ± 3.0968**e** − 008
*f*_3_	4.4415*e* − 016 ± 2.5108*e* − 016	8.9382*e* − 016 ± 8.1571*e* − 016	8.8817**e** − 016 ± 0.0000**e** − 000	2.1438*e* − 015 ± 2.6596*e* − 015
*f*_4_	0 ± 0	0 ± 0	0 ± 0	0 ± 0
*f*_5_	0 ± 0	0 ± 0	0 ± 0	0 ± 0
*f*_6_	0 ± 0	0 ± 0	0 ± 0	0 ± 0
*f*_7_	0 ± 0	0 ± 0	0 ± 0	0 ± 0
*f*_8_	0 ± 0	0 ± 0	0 ± 0	0 ± 0
*f*_9_	3.2039*e* − 015 ± 1.8147*e* − 015	2.6532**e** − 015 ± 1.3037**e** − 015	3.9092*e* − 015 ± 1.9459*e* − 015	4.3241*e* − 015 ± 2.9542*e* − 015
*f*_10_	0 ± 0	0 ± 0	0 ± 0	0 ± 0
*f*_11_	0 ± 0	0 ± 0	0 ± 0	0 ± 0
*f*_12_	5.1900*e* + 001 ± 4.3580*e* + 001	4.1625*e* + 001 ± 9.1218*e* + 000	2.6201**e** + 001 ± 8.3454**e** + 000	2.9186*e* + 001 ± 6.7990*e* + 001
*f*_13_	6.5345*e* + 001 ± 3.4082*e* + 001	5.1253*e* + 001 ± 2.3126*e* + 001	4.5502**e** + 001 ± 1.0609**e** + 001	4.8247*e* + 001 ± 1.0493*e* + 001
*f*_14_	3.0358*e* − 003 ± 3.8244*e* − 003	4.2769*e* − 003 ± 2.5513*e* − 003	2.4186**e** − 003 ± 5.3084**e** − 003	3.4354*e* − 003 ± 4.1538*e* − 003
*f*_15_	0 ± 0	0 ± 0	0 ± 0	0 ± 0
*f*_16_	9.4154*e* − 002 ± 7.1725*e* − 002	8.8757*e* − 002 ± 1.4384*e* − 002	8.8501**e** − 002 ± 1.5560**e** − 001	9.6583*e* − 002 ± 1.7236*e* − 001

**Table 4:** Results (mean ± std) of RHCSA with varying recombination rate in 16 benchmark functions with *D* = 10.

Function	0.1	0.5	0.7	1
*f*_1_	3.8119*e* − 119 ± 3.8510*e* − 119	4.0163*e* − 178 ± 0.0000*e* − 000	7.2585**e** − 195 ± 0.0000**e** − 000	3.0122*e* − 178 ± 0.0000*e* − 000
*f*_2_	2.9186*e* − 004 ± 1.1966*e* − 004	3.1115*e* − 004 ± 1.05044*e* − 004	8.9516*e* − 004 ± 3.20393*e* − 004	2.2544**e** − 004 ± 1.5452**e** − 004
*f*_3_	1.9741*e* − 015 ± 1.9767*e* − 015	1.4807*e* − 015 ± 1.9052*e* − 015	8.8817**e** − 016 ± 0.0000**e** − 000	1.4135*e* − 015 ± 1.9876*e* − 015
*f*_4_	0 ± 0	0 ± 0	0 ± 0	0 ± 0
*f*_5_	0 ± 0	0 ± 0	0 ± 0	0 ± 0
*f*_6_	0 ± 0	0 ± 0	0 ± 0	0 ± 0
*f*_7_	0 ± 0	0 ± 0	0 ± 0	0 ± 0
*f*_8_	0 ± 0	0 ± 0	0 ± 0	0 ± 0
*f*_9_	5.6305*e* − 015 ± 1.3377*e* − 015	7.9474*e* − 015 ± 2.5291*e* − 015	3.9292**e** − 015 ± 1.1420**e** − 015	4.1535*e* − 015 ± 1.2103*e* − 015
*f*_10_	3.7179*e* − 002 ± 1.8338*e* − 002	3.1530**e** − 002 ± 1.7649**e** − 002	3.8363*e* − 002 ± 3.77691*e* − 002	3.7971*e* − 002 ± 3.7825*e* − 002
*f*_11_	0 ± 0	0 ± 0	0 ± 0	0 ± 0
*f*_12_	2.3153*e* + 000 ± 1.3464*e* + 000	2.3031*e* + 000 ± 1.3465*e* + 000	1.5954*e* + 000 ± 1.5954*e* + 000	1.3685**e** + 000 ± 1.4679**e** + 000
*f*_13_	4.0817*e* + 000 ± 5.5791*e* + 000	3.8531**e** + 000 ± 2.2514**e** + 000	4.0000*e* + 000 ± 2.1213*e* + 000	4.8073*e* + 000 ± 3.4243*e* + 000
*f*_14_	5.5773**e** − 008 ± 3.6818**e** − 007	6.9679*e* − 008 ± 5.9121*e* − 007	9.8137*e* − 008 ± 7.7851*e* − 007	7.0125*e* − 008 ± 1.0013*e* − 007
*f*_15_	0 ± 0	0 ± 0	0 ± 0	0 ± 0
*f*_16_	8.6735*e* + 000 ± 8.3012*e* + 000	6.2013*e* + 000 ± 4.2016*e* + 000	5.2613**e** + 000 ± 5.0573**e** + 000	5.6012*e* + 000 ± 7.1232*e* + 000

**Table 5:** Results (mean ± std) of RHCSA with varying recombination rate in 16 benchmark functions with *D* = 30.

Function	0.1	0.5	0.7	1
*f*_1_	1.6848*e* − 121 ± 0.0000*e* − 000	1.1232*e* − 150 ± 0.0000*e* − 000	1.6994*e* − 194 ± 0.0000*e* − 000	8.4243**e** − 198 ± 0.0000**e** − 000
*f*_2_	3.3269*e* − 007 ± 8.1537*e* − 007	3.3493*e* − 007 ± 3.1302*e* − 007	2.6862**e** − 007 ± 6.0065**e** − 007	7.3295*e* − 007 ± 3.0183*e* − 007
*f*_3_	9.6645*e* − 016 ± 2.5121*e* − 016	8.9312*e* − 016 ± 2.8421*e* − 016	8.8817**e** − 016 ± 0.0000**e** − 000	2.6645*e* − 015 ± 2.7580*e* − 015
*f*_4_	0 ± 0	0 ± 0	0 ± 0	0 ± 0
*f*_5_	0 ± 0	0 ± 0	0 ± 0	0 ± 0
*f*_6_	0 ± 0	0 ± 0	0 ± 0	0 ± 0
*f*_7_	0 ± 0	0 ± 0	0 ± 0	0 ± 0
*f*_8_	0 ± 0	0 ± 0	0 ± 0	0 ± 0
*f*_9_	2.5600*e* − 015 ± 2.2178*e* − 015	2.4424**e** − 015 ± 1.2352**e** − 015	3.9092*e* − 015 ± 1.9459*e* − 015	2.5461*e* − 015 ± 2.2735*e* − 015
*f*_10_	0 ± 0	0 ± 0	0 ± 0	0 ± 0
*f*_11_	0 ± 0	0 ± 0	0 ± 0	0 ± 0
*f*_12_	4.6654*e* + 001 ± 4.1276*e* + 001	2.7521*e* + 001 ± 1.0387*e* + 000	2.6201**e** + 001 ± 8.3454**e** + 000	2.8012*e* + 001 ± 1.2293*e* + 001
*f*_13_	5.1231*e* + 001 ± 1.3024*e* + 001	4.5613*e* + 001 ± 1.3450*e* + 001	4.5502**e** + 001 ± 1.0609**e** + 001	4.7234*e* + 001 ± 1.1249*e* + 001
*f*_14_	2.6782*e* − 003 ± 3.2933*e* − 003	2.8334*e* − 003 ± 6.2013*e* − 003	2.4186**e** − 003 ± 5.3084**e** − 003	3.0187*e* − 003 ± 8.7612*e* − 003
*f*_15_	0 ± 0	0 ± 0	0 ± 0	0 ± 0
*f*_16_	8.7011*e* − 002 ± 5.6745*e* − 002	8.7329*e* − 002 ± 9.2581*e* − 002	8.8501*e* − 002 ± 1.5560*e* − 001	6.0112**e** − 002 ± 2.5832**e** − 002

**Table 6:** Results of traditional clonal selection algorithm and RHCSA when *D* = 10.

ALGs	Clonal selection algorithm (CSA)		CSA with recombination		RHCSA	
	Mean	Std	Mean	Std	Mean	Std
*f*_1_	5.0414*e* − 008	4.4098*e* − 008	6.9076*e* − 056	9.5731*e* − 055	7.2585**e** − 195	0
*f*_2_	5.5057*e* + 000	2.7914*e* + 000	4.5314*e* − 003	4.7750*e* − 002	8.9516**e** − 004	3.2039*e* − 004
*f*_3_	2.8532*e* − 003	8.5712*e* − 003	2.3976*e* − 015	5.7314*e* − 015	8.8817**e** − 016	0
*f*_4_	2.6092*e* − 002	1.9092*e* − 002	0	0	0	0
*f*_5_	1.2569*e* − 002	4.4216*e* − 003	0	0	0	0
*f*_6_	8.4141*e* + 000	2.4848*e* + 000	0	0	0	0
*f*_7_	5.7681*e* + 000	1.3571*e* + 000	0	0	0	0
*f*_8_	3.6436*e* + 002	9.7648*e* + 001	0	0	0	0
*f*_9_	1.0619*e* + 000	7.9728*e* − 001	1.3592*e* − 008	1.0975*e* − 008	3.9292**e** − 015	1.1420*e* − 015
*f*_10_	4.2974*e* − 001	1.0264*e* − 001	2.5301*e* − 001	1.1356*e* − 001	1.6937**e** − 002	1.6091*e* − 002
*f*_11_	6.0837*e* + 000	1.5496*e* + 000	5.2403*e* − 008	1.2560*e* − 008	0	0
*f*_12_	4.2193*e* + 001	5.4173*e* + 000	3.6021*e* + 001	1.2541*e* + 001	2.7006**e** + 000	1.5954*e* + 000
*f*_13_	4.3672*e* + 001	5.5570*e* + 000	2.3702*e* + 001	2.5091*e* + 001	4.0000**e** + 000	2.1213*e* + 000
*f*_14_	1.9305*e* + 003	2.3358*e* + 002	2.7201*e* − 003	6.1034*e* − 003	9.8137**e** − 008	7.7851*e* − 007
*f*_15_	4.5354*e* + 001	3.2450*e* + 001	1.0276*e* − 033	1.8734*e* − 033	0	0
*f*_16_	5.1701*e* + 001	1.7347*e* + 001	5.2613*e* + 000	5.0573*e* + 000	5.2613**e** + 000	5.0573*e* + 000

**Table 7:** Results of traditional clonal selection algorithm and RHCSA when *D* = 30.

ALGs	Clonal selection algorithm (CSA)		CSA with recombination		RHCSA	
	Mean	Std	Mean	Std	Mean	Std
*f*_1_	6.4595*e* − 002	2.9504*e* − 002	1.4668*e* − 105	3.0015*e* − 105	1.6994**e** − 194	0
*f*_2_	2.7748*e* + 001	2.1866*e* + 000	1.8524*e* − 001	2.6531*e* − 001	2.6862**e** − 007	6.0065*e* − 007
*f*_3_	3.1589*e* − 001	1.8716*e* − 001	1.9187**e** − 017	3.8302*e* − 017	8.8817*e* − 016	0
*f*_4_	1.6746*e* − 001	4.3211*e* − 002	0	0	0	0
*f*_5_	3.6752*e* + 001	4.6907*e* + 000	0	0	0	0
*f*_6_	3.6752*e* + 001	4.6907*e* + 000	0	0	0	0
*f*_7_	2.3935*e* + 001	2.5226*e* + 000	0	0	0	0
*f*_8_	1.6279*e* + 003	1.7813*e* + 002	0	0	0	0
*f*_9_	3.8700*e* + 000	3.2556*e* − 001	1.8245*e* − 005	1.3675*e* − 005	3.9092**e** − 015	1.9459*e* − 015
*f*_10_	8.3333*e* − 001	7.5256*e* − 002	2.9176*e* − 028	1.3613*e* − 028	0	0
*f*_11_	3.3702*e* + 001	2.7162*e* + 000	1.1680*e* − 030	1.9130*e* − 030	0	0
*f*_12_	2.5929*e* + 002	1.4658*e* + 001	4.3898*e* + 001	2.0666*e* + 000	2.6201**e** + 001	8.3454*e* + 000
*f*_13_	2.5762*e* + 002	2.1376*e* + 001	5.4715*e* + 001	1.7261*e* + 001	4.5502**e** + 001	1.0609*e* + 001
*f*_14_	8.7875*e* + 003	3.2136*e* + 002	2.5296*e* − 002	1.6225*e* − 002	2.4186**e** − 003	5.3084*e* − 003
*f*_15_	4.4892*e* + 001	9.5942*e* + 000	3.3097*e* − 033	1.6468*e* − 033	0	0
*f*_16_	3.9889*e* + 001	4.3023*e* + 000	2.5177*e* − 001	1.1168*e* − 001	8.8501**e** − 002	1.5560*e* − 001

**(a):** 

ALGs	*f*_1_	*f*_2_	*f*_3_	*f*_4_
RHCSA	7.2585*e* − 195 ± 0.000*e* − 000	8.9516*e* − 004 ± 3.2039*e* − 004	8.8817**e** − 016 ± 0.000**e** − 000	0 ± 0
HLCSA	4.2228*e* − 053 ± 9.9892*e* − 053	3.9087**e** − 028 ± 6.6496**e** − 028	2.5757*e* − 015 ± 4.8648*e* − 016	0 ± 0
BCSA	1.1694*e* − 037 ± 1.3065*e* − 037	1.8521*e* − 001 ± 7.2808*e* − 001	2.6645*e* − 015 ± 0.0000*e* − 000	1.4146*e* − 001 ± 1.3744*e* − 001
OXDE	4.5059*e* − 056 ± 7.4966*e* − 056	1.0265*e* − 026 ± 2.0786*e* − 026	2.0724*e* − 015 ± 1.3467*e* − 015	9.9330*e* − 004 ± 1.0673*e* − 002
SaDE	1.4451*e* − 176 ± 0.0000*e* − 000	2.0249*e* + 000 ± 7.4832*e* − 001	5.0330*e* − 015 ± 1.1109*e* − 015	1.8074*e* − 003 ± 3.8251*e* − 003
GL-25	1.0771**e** − 321 ± 0.0000**e** − 000	2.0956*e* + 000 ± 6.3579*e* − 001	2.7830*e* − 015 ± 1.4703*e* − 015	1.2134*e* − 002 ± 1.0199*e* − 002
CLPSO	1.8154*e* − 041 ± 3.0360*e* − 041	2.1490*e* + 000 ± 1.2450*e* + 000	3.9672*e* − 015 ± 1.7413*e* − 015	7.4577*e* − 006 ± 2.1864*e* − 005

**(b):** 

ALGs	*f*_5_	*f*_6_	*f*_7_	*f*_8_

RHCSA	0 ± 0	0 ± 0	0 ± 0	0 ± 0
HLCSA	0 ± 0	0 ± 0	0 ± 0	0 ± 0
BCSA	0 ± 0	0 ± 0	0 ± 0	0 ± 0
OXDE	0 ± 0	6.6331*e* − 002 ± 2.5243*e* − 001	5.6667*e* − 001 ± 7.2793*e* − 001	0 ± 0
SaDE	0 ± 0	0 ± 0	0 ± 0	0 ± 0
GL-25	7.3315*e* − 007 ± 2.2405*e* − 006	1.9633*e* + 000 ± 1.1774*e* + 000	5.6336*e* + 000 ± 1.2724*e* + 000	2.8952*e* + 002 ± 1.9959*e* + 002
CLPSO	0 ± 0	0 ± 0	0 ± 0	0 ± 0

**(c):** 

ALGs	*f*_9_	*f*_10_	*f*_11_	*f*_12_

RHCSA	3.9292*e* − 015 ± 1.1420*e* − 015	1.6937*e* − 002 ± 1.6091*e* − 002	0 ± 0	1.5954**e** + 000 ± 1.5954**e** + 000
HLCSA	3.5527*e* − 015 ± 0.0000*e* − 000	2.8802*e* − 002 ± 1.9129*e* − 002	0 ± 0	4.2783*e* + 000 ± 2.0095*e* + 000
BCSA	3.5527*e* − 015 ± 0.0000*e* − 000	3.2715*e* − 001 ± 1.7104*e* − 001	0 ± 0	6.2881*e* + 001 ± 1.6334*e* + 001
OXDE	3.1974*e* − 015 ± 1.0840*e* − 015	4.9045*e* − 001 ± 2.7411*e* − 002	0 ± 0	3.7808*e* + 000 ± 1.9094*e* + 000
SaDE	9.4739**e** − 016 ± 1.5979**e** − 015	1.3704*e* − 002 ± 1.6048*e* − 002	0 ± 0	3.9135*e* + 000 ± 1.4295*e* + 000
GL-25	3.5527*e* − 015 ± 0.0000*e* − 000	1.0545**e** − 002 ± 1.0858**e** − 002	2.1771*e* − 004 ± 4.7010*e* − 004	3.3619*e* + 000 ± 2.2861*e* + 000
CLPSO	3.8606*e* − 014 ± 5.8665*e* − 014	2.8592*e* − 002 ± 1.5307*e* − 002	1.4403*e* − 002 ± 1.2235*e* − 002	4.1634*e* + 000 ± 9.0362*e* − 001

**(d):** 

ALGs	*f*_13_	*f*_14_	*f*_15_	*f*_16_

RHCSA	4.0000*e* + 000 ± 2.1213*e* + 000	9.8137*e* − 008 ± 7.7851*e* − 007	0 ± 0	5.2613*e* + 000 ± 5.0573*e* + 000
HLCSA	4.2442*e* + 000 ± 2.6469*e* + 000	0 ± 0	0 ± 0	4.6177**e** − 001 ± 8.1247**e** − 001
BCSA	6.5935*e* + 001 ± 1.0618*e* + 001	2.6391*e* + 002 ± 7.9682*e* + 001	4.3387*e* − 031 ± 6.5797*e* − 031	8.7291*e* + 000 ± 1.7669*e* + 001
OXDE	3.0956*e* + 000 ± 1.1245*e* + 000	1.5792*e* + 001 ± 6.7669*e* + 001	0 ± 0	1.0047*e* + 001 ± 3.0498*e* + 001
SaDE	3.9534*e* + 000 ± 1.9500*e* + 000	2.0681*e* + 002 ± 8.6302*e* + 001	0 ± 0	1.8019*e* + 001 ± 3.7308*e* + 001
GL-25	7.3657*e* + 000 ± 2.2262*e* + 000	5.2254*e* + 002 ± 1.7963*e* + 002	0 ± 0	9.0000*e* + 001 ± 3.0513*e* + 001
CLPSO	2.0254**e** + 000 ± 1.0621**e** + 000	3.1281*e* + 002 ± 1.5723*e* + 002	2.3104*e* − 002 ± 6.5636*e* − 002	6.0233*e* + 000 ± 4.0698*e* + 000

**(a):** 

ALGs	*f*_1_	*f*_2_	*f*_3_	*f*_4_
RHCSA	1.6994*e* − 194 ± 0.0000*e* − 000	7.4820*e* − 001 ± 3.0968*e* − 001	8.8817**e** − 016 ± 8.8817**e** − 016	0 ± 0
HLCSA	7.1289*e* − 066 ± 2.0169*e* − 065	1.1617**e** − 015 ± 5.5448**e** − 015	2.6645*e* − 015 ± 0.0000*e* − 000	0 ± 0
BCSA	2.9665*e* − 025 ± 8.4182*e* − 025	1.9018*e* + 001 ± 2.6925*e* + 000	1.5614*e* − 013 ± 3.9345*e* − 013	0 ± 0
OXDE	4.8545*e* − 059 ± 1.2064*e* − 058	2.6577*e* − 001 ± 1.0114*e* + 000	2.6645*e* − 001 ± 0.0000*e* − 000	2.8730*e* − 003 ± 5.6727*e* − 003
SaDE	9.1236*e* − 150 ± 4.4538*e* − 149	2.1973*e* + 001 ± 1.0132*e* + 000	7.7383*e* − 001 ± 6.0009*e* − 001	1.1999*e* − 002 ± 1.9462*e* − 002
GL-25	5.3539**e** − 228 ± 0.0000**e** − 000	2.0832*e* + 001 ± 8.6842*e* − 001	8.4969*e* − 014 ± 1.7664*e* − 013	9.7959*e* − 015 ± 3.2264*e* − 014
CLPSO	1.9761*e* − 029 ± 1.5041*e* − 029	1.7605*e* + 001 ± 3.6364*e* + 000	1.8415*e* − 014 ± 3.0495*e* − 015	1.1102*e* − 016 ± 3.2467*e* − 016

**(b):** 

ALGs	*f*_5_	*f*_6_	*f*_7_	*f*_8_

RHCSA	0 ± 0	0 ± 0	0 ± 0	0 ± 0
HLCSA	0 ± 0	0 ± 0	0 ± 0	0 ± 0
BCSA	0 ± 0	0 ± 0	0 ± 0	0 ± 0
OXDE	1.6214*e* − 003 ± 6.5408*e* − 003	9.4189*e* − 000 ± 2.0859*e* − 000	1.5100*e* + 001 ± 2.9868*e* + 000	3.9479*e* + 000 ± 2.1624*e* + 001
SaDE	9.5195*e* − 002 ± 1.5798*e* − 001	8.6230*e* − 001 ± 8.9502*e* − 001	6.3333*e* − 001 ± 7.6489*e* − 001	3.9479*e* + 001 ± 6.4747*e* + 001
GL-25	7.1724 − *e*004 ± 4.8027*e* − 004	2.3030*e* + 001 ± 8.4952*e* + 000	3.9096*e* + 001 ± 2.2071*e* + 001	3.5030*e* + 003 ± 6.8004*e* + 000
CLPSO	0 ± 0	0 ± 0	8.7634*e* − 015 ± 1.3333*e* − 014	0 ± 0

**(c):** 

ALGs	*f*_9_	*f*_10_	*f*_11_	*f*_12_

RHCSA	3.9092*e* − 015 ± 1.9459*e* − 015	0 ± 0	0 ± 0	2.6201*e* + 001 ± 8.3454*e* + 000
HLCSA	3.5527**e** − 015 ± 0.0000**e** − 000	0 ± 0	0 ± 0	2.5471*e* + 001 ± 6.7663*e* + 000
BCSA	1.3086*e* − 013 ± 3.4341*e* − 013	1.2273*e* − 002 ± 2.2389*e* − 002	1.8516*e* − 014 ± 4.8488*e* − 014	3.2115*e* + 001 ± 9.0135*e* + 000
OXDE	3.5527**e** − 015 ± 0.0000**e** − 000	1.5612*e* − 003 ± 3.2032*e* − 003	1.4210*e* − 001 ± 2.3765*e* − 001	1.6549**e** + 001 ± 4.4609**e** + 000
SaDE	1.1708*e* + 000 ± 6.5356*e* − 001	1.3096*e* − 002 ± 2.6787*e* − 002	2.0504*e* + 000 ± 9.0887*e* − 001	2.5050*e* + 001 ± 6.6415*e* + 000
GL-25	1.1416*e* − 013 ± 1.6841*e* − 013	4.2040*e* − 015 ± 5.5414*e* − 015	5.6795*e* − 003 ± 2.6720*e* − 003	2.9464*e* + 001 ± 2.2594*e* + 001
CLPSO	1.4501*e* − 007 ± 7.0645*e* − 007	4.4152*e* − 007 ± 7.4331*e* − 007	1.7977*e* + 000 ± 6.1941*e* − 001	4.6287*e* + 001 ± 5.7149*e* + 000

**(d):** 

ALGs	*f*_13_	*f*_14_	*f*_15_	*f*_16_

RHCSA	4.5502*e* + 001 ± 1.0609*e* + 001	2.4186**e** − 003 ± 5.3084**e** − 003	0 ± 0	8.8501**e** − 002 ± 1.5560**e** − 001
HLCSA	4.7609*e* + 001 ± 1.5104*e* + 001	1.0663*e* + 003 ± 5.2886*e* + 002	0 ± 0	3.2212*e* + 000 ± 1.0133*e* + 000
BCSA	2.6912*e* + 001 ± 1.2152*e* + 001	2.6533*e* + 003 ± 5.3339*e* + 002	2.0259*e* − 023 ± 6.3626*e* − 023	1.8053*e* + 001 ± 1.8282*e* + 001
OXDE	1.7959**e** + 001 ± 5.1559**e** + 000	4.3428*e* + 001 ± 8.8341*e* + 001	3.3333*e* + 000 ± 1.8257*e* + 001	2.5992*e* + 000 ± 5.6332*e* − 001
SaDE	2.2788*e* + 001 ± 6.6879*e* + 000	2.4742*e* + 003 ± 5.9013*e* + 002	1.1833*e* − 031 ± 2.1659*e* − 031	1.0208*e* + 001 ± 1.7851*e* + 001
GL-25	9.6862*e* + 001 ± 4.0914*e* + 001	3.2335*e* + 003 ± 5.9871*e* + 002	2.7878*e* − 028 ± 1.1207*e* − 027	5.2187*e* + 001 ± 2.1654*e* + 001
CLPSO	4.0333*e* + 001 ± 7.5039*e* + 000	2.6321*e* + 003 ± 3.3553*e* + 002	8.2952*e* − 005 ± 3.3295*e* − 004	7.9983*e* + 000 ± 1.6728*e* + 000

## References

[1] Liang J. J., Qin A. K., Suganthan P. N., Baskar S. (2006). Comprehensive learning particle swarm optimizer for global optimization of multimodal functions. *IEEE Transactions on Evolutionary Computation*.

[2] Jordehi A. R. (2015). Enhanced leader PSO (ELPSO): a new PSO variant for solving global optimisation problems. *Applied Soft Computing*.

[3] Jordehi A. R. (2015). Seeker optimisation (human group optimisation) algorithm with chaos. *Journal of Experimental and Theoretical Artificial Intelligence*.

[4] Yang X.-S., Gandomi A. H. (2012). Bat algorithm: a novel approach for global engineering optimization. *Engineering Computations*.

[5] Jordehi A. R. (2015). Brainstorm optimisation algorithm (BSOA): an efficient algorithm for finding optimal location and setting of FACTS devices in electric power systems. *International Journal of Electrical Power & Energy Systems*.

[6] Heidari A. A., Abbaspour R. A., Jordehi A. R. (2015). An efficient chaotic water cycle algorithm for optimization tasks. *Neural Computing & Applications*.

[7] Boussaïd I., Lepagnot J., Siarry P. (2013). A survey on optimization metaheuristics. *Information Sciences*.

[8] Hart E., McEwan C., Timmis J., Hone A. (2011). Advances in artificial immune systems. *Evolutionary Intelligence*.

[9] Xu Y., Jiang Y., Deng W. An novel immune genetic algorithm and its application in WWTP.

[10] Daoudi R., Djemal K., Benyettou A. Improving cells recognition by local database categorization in Artificial Immune System algorithm.

[11] Lu Z., Pei G., Liu B., Liu Z. Hardware implementation of negative selection algorithm for malware detection.

[12] Pavone M., Narzisi G., Nicosia G. (2012). Clonal selection: an immunological algorithm for global optimization over continuous spaces. *Journal of Global Optimization*.

[13] Liu R., Jiao L., Zhang X., Li Y. (2012). Gene transposon based clone selection algorithm for automatic clustering. *Information Sciences*.

[14] Batista L. D. S., Guimarães F. G., Ramirez J. A. (2009). A distributed clonal selection algorithm for optimization in electromagnetics. *IEEE Transactions on Magnetics*.

[15] Zheng J., Chen Y., Zhang W. (2010). A survey of artificial immune applications. *Artificial Intelligence Review*.

[16] de Castro L. N., Timmis J. (2002). *Artificial Immune Systems: A New Computational Intelligence Approach*.

[17] Cruz-Cortés N. (2009). Handling constraints in global optimization using artificial immune systems: a survey. *Constraint-Handling in Evolutionary Optimization*.

[18] Mezura-Montes E., Coello Coello C. A. (2011). Constraint-handling in nature-inspired numerical optimization: past, present and future. *Swarm and Evolutionary Computation*.

[19] Dasgupta D., Yu S., Nino F. (2011). Recent advances in artificial immune systems: models and applications. *Applied Soft Computing Journal*.

[20] de Castro L. N., von Zuben F. J. (2002). Learning and optimization using the clonal selection principle. *IEEE Transactions on Evolutionary Computation*.

[21] Cutello V., Nicosia G., Pavone M. Real coded clonal selection algorithm for unconstrained global optimization using a hybrid inversely proportional hypermutation operator.

[22] Jansen T., Zarges C. (2011). Analyzing different variants of immune inspired somatic contiguous hypermutations. *Theoretical Computer Science*.

[23] Khilwani N., Prakash A., Shankar R., Tiwari M. K. (2008). Fast clonal algorithm. *Engineering Applications of Artificial Intelligence*.

[24] Lu H., Yang J. An improved clonal selection algorithm for job shop scheduling.

[25] Liu X., Shi L., Chen R., Chen H. A novel clonal selection algorithm for global optimization problems.

[26] De Castro L. N., Timmis J. An artificial immune network for multimodal function optimization.

[27] Li Z., Zhang Y., Tan H.-Z. (2011). IA-AIS: an improved adaptive artificial immune system applied to complex optimization problems. *Applied Soft Computing*.

[28] Li Z., He C., Tan H.-Z. (2013). AINet-SL: artificial immune network with social learning and its application in FIR designing. *Applied Soft Computing Journal*.

[29] Li Z., Li J., Zhou J. An improved artificial immune network for multimodal function optimization.

[30] Coelho G. P., Zuben F. J. V. A concentration-based artificial immune network for continuous optimization.

[31] Coelho G. P., De Franca F. O., Zuben F. J. V. A concentration-based artificial immune network for combinatorial optimization.

[32] Coelho G. P., Zuben F. J. V. A concentration-based artificial immune network for multi-objective optimization.

[33] Yildiz A. R. (2009). An effective hybrid immune-hill climbing optimization approach for solving design and manufacturing optimization problems in industry. *Journal of Materials Processing Technology*.

[34] Gong M., Zhang L., Jiao L., Ma W. Differential immune clonal selection algorithm.

[35] Gong M., Jiao L., Liu F., Ma W. (2010). Immune algorithm with orthogonal design based initialization, cloning, and selection for global optimization. *Knowledge & Information Systems*.

[36] Gong M., Jiao L., Du H., Bo L. (2008). Multi-objective immune algorithm with non-dominated neighbor-based selection. *Evolutionary Computation*.

[37] Shang R., Jiao L., Liu F., Ma W. (2012). A novel immune clonal algorithm for MO problems. *IEEE Transactions on Evolutionary Computation*.

[38] Gong M., Jiao L., Zhang L. (2010). Baldwinian learning in clonal selection algorithm for optimization. *Information Sciences*.

[39] Gong M., Jiao L., Yang J., Liu F. (2010). Lamarckian learning in clonal selection algorithm for numerical optimization. *International Journal on Artificial Intelligence Tools*.

[40] Peng Y., Lu B. (2015). Hybrid learning clonal selection algorithm. *Information Sciences*.

[41] De Castro L. N., Zuben F. J. V. (1999). Artificial immune systems: part I—basic theory and applications.

[42] Bersini H. (2002). The immune and the chemical crossover. *IEEE Transactions on Evolutionary Computation*.

[43] Qi Y., Hou Z., Yin M., Sun H., Huang J. (2015). An immune multi-objective optimization algorithm with differential evolution inspired recombination. *Applied Soft Computing Journal*.

[44] Liu R., Ma C. L., He F., Ma W. P., Jiao L. C. (2014). Reference direction based immune clone algorithm for many-objective optimization. *Frontiers in Computer Science*.

[45] Gao W.-F., Liu S.-Y., Huang L.-L. (2013). A novel artificial bee colony algorithm based on modified search equation and orthogonal learning. *IEEE Transactions on Cybernetics*.

[46] Liang J. J., Suganthan P. N., Deb K. Novel composition test functions for numerical global optimization.

[47] Wang Y., Cai Z., Zhang Q. (2012). Enhancing the search ability of differential evolution through orthogonal crossover. *Information Sciences*.

[48] Qin A. K., Huang V. L., Suganthan P. N. (2009). Differential evolution algorithm with strategy adaptation for global numerical optimization. *IEEE Transactions on Evolutionary Computation*.

[49] García-Martínez C., Lozano M., Herrera F., Molina D., Sánchez A. M. (2008). Global and local real-coded genetic algorithms based on parent-centric crossover operators. *European Journal of Operational Research*.

